# Associations between volatile flavour compounds and microbial communities during the fermentation of traditional mould-fermented Sufu

**DOI:** 10.1016/j.fochx.2026.104552

**Published:** 2026-10-03

**Authors:** Xiangcheng Lu, Xiaofeng Niu, Zhiming Wu, Hongmei Zhao, Yanjun Yang, Xiaoli Ma, Shengli Shi, Huihui Wang, Bianfang Hu, Aiguo Luo

**Affiliations:** a Department of Biological Science and Technology, Jinzhong University, Jinzhong 030619, China; b College of Food Science and Engineering, Shanxi Agricultural University, Jinzhong 030801, China; c Shanxi Liuweizhai Holdings Group Co., Ltd, Taiyuan 030400, China; d Shanxi Center of Technology Innovation for Compound Seasonings, Jinzhong University, Jinzhong 030801, China; e Jinzhong Normal College, Jinzhong 030600, China

**Keywords:** Sufu, Volatile flavour compounds, Relative odour activity value (ROAV), Microbial community dynamics

## Abstract

Traditional mould-fermented sufu develops its distinctive flavour through interplay between volatile compound formation and microbial succession, yet the underlying microbial basis remains unclear. This study profiled volatile compounds and microbial dynamics across six fermentation stages (days 1–15) using HS-GC–MS, ROAV analysis, and high-throughput sequencing. Of 105 volatile compounds detected, ROAV identified 13 potential key aroma-active compounds, including isoamyl alcohol, linalool, heptanal, d-limonene, and 2-pentylfuran, with key esters accumulating in later stages. Fungal richness exceeded bacterial richness in early-to-middle stages, and fungal Shannon diversity exceeded bacterial diversity from Day 3 onward. Ascomycota, *Thermomyces*, and *Thermoascus* dominated fungal communities, while Proteobacteria and *Acinetobacter* dominated bacterial communities. Pearson analysis showed Ascomycota, *Thermoascus*, *Thermomyces*, *Pichia*, and Proteobacteria positively correlated with these compounds, whereas Mucoromycota and Firmicutes showed negative associations. These findings suggest potential microbial associations with flavour formation, providing preliminary evidence for flavour regulation and quality improvement of traditional sufu.

## Highlights

• HS-GC–MS identified 105 volatile compounds, with 13 key aroma-active compounds screened by ROAV analysis.

## 1 Introduction

Traditional mould-fermented sufu is one of the most representative traditional fermented soybean foods in China. It is typically produced by inoculation with mould starters, such as Mucor species, followed by pre-fermentation and post-salting ripening processes that contribute to its distinctive flavour and texture characteristics. Compared with industrially fermented products, traditionally produced sufu retains a more complex microbial ecosystem and consequently develops a richer flavour profile. The formation of sufu flavour is a complex biochemical process involving the continuous degradation and transformation of soybean proteins, lipids, and carbohydrates by microorganisms, leading to the generation of alcohols, aldehydes, esters, and other flavour-active compounds (Xie et al., 2024). However, the relationships between microbial succession and flavour formation during traditional mould-fermented sufu fermentation require further investigation.

Considerable progress has been made in understanding flavour formation mechanisms in fermented soybean products. Previous studies have investigated the microbial community structures and flavour characteristics of gray sufu (Xie et al., 2024), red sufu (Li et al., 2022), and stinky tofu (Luo et al., 2025), suggesting that microbial succession may be closely associated with the formation of volatile flavour compounds. In addition, studies on traditional fermented foods such as soy sauce (Wu et al., 2023), vinegar (Ye et al., 2024), and fermented alcoholic beverages (Zheng et al., 2023) have revealed significant associations between changes in microbial community composition and the formation of key flavour compounds, including alcohols, aldehydes, and esters. Previous studies on sufu flavour have mainly focused on the identification and sensory contribution of aroma-active compounds. Aroma-active compounds in Guilin Huaqiao white sufu were characterized and shown to play important roles in aroma perception and flavour enhancement (He et al., 2020). However, these studies primarily addressed the composition and sensory significance of volatile compounds, whereas the microbial ecological basis underlying the dynamic changes in volatile flavour compounds during traditional mould-fermented sufu fermentation remains insufficiently understood.

With the advancement of analytical technologies, headspace gas chromatography–mass spectrometry (HS-GC–MS) and high-throughput sequencing have become important tools for characterising flavour compounds and microbial succession in fermented foods (Li et al., 2022). Among these approaches, the Relative Odour Activity Value (ROAV) method integrates odour thresholds and relative abundances of volatile compounds to evaluate their relative contributions to the overall aroma profile and has been widely applied as a screening approach for identifying key aroma-active compounds in fermented foods (Wang et al., 2025). Recent advances in fermented food microbiome research have further highlighted the importance of multidimensional characterization of microbial ecosystems for understanding fermentation quality and flavour development (Wu et al., 2026). These developments suggest that combining microbial ecological analysis with flavour characterization provides a promising approach for understanding the formation of characteristic flavours in traditional fermented foods.

Therefore, this study used traditionally produced mould-fermented sufu as the research subject. Samples were collected at different fermentation stages (1, 3, 6, 9, 12, and 15 d). HS-GC–MS was employed to analyse changes in volatile flavour compounds, and the ROAV method was used to identify potential key aroma-active compounds. High-throughput sequencing was further applied to characterise bacterial and fungal community succession, and association analysis was conducted to explore potential associations between microbial communities and potential key aroma-active compounds. The findings of this study contribute to a better understanding of potential microbial associations involved in flavour development in traditional sufu and provide a theoretical basis for flavour regulation, process optimisation, and quality improvement of traditional fermented soybean products.

## 2 Materials and methods

### 2.1 Experimental materials and instruments

The mould starter culture (composed of rice flour, wheat bran, and Mucor spp.) was purchased from Hubei Chuzhai Fermentation Products Co., Ltd. Firm tofu, chilli powder, and Sichuan pepper powder were sourced locally. Chinese liquor (Erguotou, 42% *v*/v alcohol content, light-aroma type) was also sourced locally. Sodium chloride (analytical grade; Tianjin Damao Chemical), agarose (Shanghai Bolsen Biotechnology), 5× TBE buffer, ethidium bromide (both Beijing Solarbio), and DNA marker (Takara Bio) were used for electrophoresis. Key instruments included a 7697 A-8860-5977C headspace GC–MS system (Agilent Technologies), a DYCZ-21 PCR thermal cycler (Dongsheng Innovation Biotechnology), a gel imaging system (Hangzhou Shenhua), and a DYY-6B electrophoresis power supply (Beijing Liuyi Instrument Factory).

### 2.2 Experimental methods

#### 2.2.1 Collection of fermented Sufu samples at different fermentation stages

Following Li's method (Li, 2008) with slight modifications, the tofu was cut into uniform cubes of 3 cm × 3 cm × 3 cm, steamed in a pot for 5 min, and then allowed to cool. Subsequently, the tofu cubes were immersed in a 9% starter culture suspension for 3 s, ensuring that all six sides of each cube were evenly coated. The inoculated tofu cubes were then placed on bamboo trays and fermented at 20 °C for 3 d to complete the primary fermentation. Following this, under sterile conditions, the mycelium covering the surface of the tofu cubes was gently wiped to form a uniform mould film and to ensure the cubes could be easily separated. Chilli powder, Sichuan pepper powder, and salt were mixed in a ratio of 1:5:2. A volume of 100 mL of Chinese liquor (Baijiu) was taken. The tofu cubes that had completed the primary fermentation were sprayed with the liquor, uniformly coated with the mixed seasoning, and then packed into bottles for a subsequent 15 d secondary fermentation to obtain the final samples.

The above sample preparation procedure (including tofu cube preparation, inoculation, primary fermentation, and secondary fermentation) was independently performed in triplicate using three separate batches of raw materials, yielding three biological replicates. Within each batch, all tofu cubes were prepared, inoculated, and fermented under identical, standardised conditions, ensuring a high degree of uniformity among cubes within the same batch. At each designated fermentation stage (days 1, 3, 6, 9, 12, and 15), one whole piece of sufu was aseptically removed from the batch using sterilised chopsticks to serve as the representative sample for that stage, while minimising disturbance and contamination of the remaining pieces. Each retrieved sample was immediately snap-frozen in liquid nitrogen and stored at −80 °C until all six stages had been sampled, after which the samples were analysed together for volatile compound and microbial community profiling.

#### 2.2.2 Analysis of flavour compounds in Sufu at different fermentation stages by HS-GC–MS

Sample pretreatment: A single piece of Sufu was ground into a paste. An aliquot of 3 g of the paste was placed into a 20 mL headspace vial, and 3 mL of saturated sodium chloride solution was added. The vial was then sealed and allowed to equilibrate prior to analysis.

Headspace conditions: The sample vial heating temperature was set at 110 °C, the loop temperature at 120 °C, and the transfer line temperature at 130 °C. The sample vial equilibration time was 10 min.

GC–MS conditions: A non-polar HP-5MS capillary column (30 m × 0.25 mm × 0.25 μm) was employed. Helium (purity >99.999%) was used as the carrier gas at a flow rate of 1.0 mL/min. The injector temperature was 250 °C, and the split ratio was 5:1. The oven temperature programme was as follows: the initial temperature was held at 40 °C for 3 min, then increased to 100 °C at a rate of 5 °C/min, subsequently raised to 220 °C at a rate of 6 °C/min and held for 10 min. A post-run step was performed at 250 °C for 5 min. The mass spectrometer was operated in electron ionisation (EI) mode with an electron energy of 70 eV. The ion source temperature was 120 °C, and the MS quadrupole temperature was 150 °C. A solvent delay of 3 min was applied. Mass spectra were scanned over a range of *m*/*z* 35–600.

The volatile compounds were identified using the instrument-supported Qualitative Analysis software in combination with the NIST23 mass spectral library. Semi-quantitative analysis was performed using the GC–MS area normalisation method, and the relative content of each volatile compound was expressed as the percentage of its peak area relative to the total peak area (Le et al., 2025).

#### 2.2.3 Identification of potential key aroma-active compounds in Sufu

The relative odour activity value (ROAV) method was employed to screen potential key aroma-active compounds in sufu samples at different fermentation stages (Li et al., 2026). The ROAV for each volatile flavour compound (A) was calculated according to Eq. (1–1) (Zhang et al., 2022). Since the volatile compounds were semi-quantified using the GC–MS area normalisation method, the calculated ROAV values were used to estimate the relative contribution of aroma compounds rather than their absolute aroma impact.

(1–1) $$\text{ROAVA}\approx 100\times \left(\mathrm{CA}/\text{Cstan}\right)\times \left(\text{Tstan}/\mathrm{TA}\right)$$

In Eq. (1–1), CA represents the relative content of each individual volatile flavour compound; Cstan represents the relative content of the volatile flavour compound that contributes most significantly to the overall flavour profile of the sample; TA is the odour threshold corresponding to each individual volatile flavour compound; and Tstan is the odour threshold of the volatile flavour compound that contributes most significantly to the overall flavour profile of the sample.

#### 2.2.4 High-throughput sequencing

Using the extracted genomic DNA as a template, the bacterial universal primers (F:ACTCCTACGGGAGGCAGCA; R:GGACTACHVGGGTWTCTAAT) and fungal universal primers (F:CTTGGTCATTTAGAGGAAGTAA; R:GCTGCGTTCTTCATCGATGC) were employed. PCR amplification of the V3 + V4 region of the bacterial 16S rRNA gene and the fungal ITS1 region was performed, and the corresponding fragments were obtained. High-throughput sequencing (paired-end) was conducted by Beijing Biomarker Technologies Co., Ltd. to analyse the community composition of bacteria and fungi in the samples, respectively.

Sequencing libraries were constructed by PCR amplification of the target regions using primers with sequencing adapters attached, followed by purification, quantification, and normalisation of the PCR products. Library quality was assessed prior to sequencing, and qualified libraries were sequenced on an Illumina NovaSeq 6000 platform (paired-end). Raw image data generated by the sequencer were converted into raw sequencing reads via base calling and stored in FASTQ format. Raw reads were quality-filtered using Trimmomatic (v0.33), and primer sequences were identified and removed using Cutadapt (v1.9.1) to obtain clean reads. Denoising, paired-end read merging, and chimera removal were performed using the DADA2 plugin within QIIME2 (v2020.6) to generate non-chimeric reads. Operational taxonomic units (OTUs) were subsequently clustered at a 97% sequence similarity threshold using USEARCH. Taxonomic annotation of representative OTU sequences was performed using a naïve Bayesian classifier combined with alignment-based matching, against the SILVA database for bacterial 16S rRNA sequences and the UNITE database for fungal ITS sequences. Community composition tables at each taxonomic level (phylum, class, order, family, genus, and species) were generated using QIIME2, and community structure plots were produced using R. Relative abundance tables were generated directly from this pipeline without additional rarefaction.

### 2.3 Data analysis

WPS Office was used to compile the relative contents of each volatile compound. Statistical analysis of the experimental data and the generation of figures were performed using Origin 2021. Microbial diversity data were analysed and visualised graphically on the Biomarker Cloud Platform. Pearson correlation analysis was performed to evaluate the potential associations between microbial communities and potential key aroma-active compounds. Although compositional-data approaches (e.g., CLR transformation and SparCC) may provide more robust estimates, Pearson correlation analysis was selected because it has been widely applied in fermented food microbiome–flavour association studies.

## 3 Results and analysis

### 3.1 Analysis of changes in volatile flavour compounds in Sufu at different fermentation stages by HS-GC–MS

Volatile flavour compounds in traditional mould-fermented sufu at different fermentation stages were analysed by HS-GC–MS, and the results are shown in Fig. 1. As shown in Fig. 1a, the chromatographic peak intensities and compositions varied considerably across fermentation stages, indicating dynamic changes in volatile flavour compounds throughout the fermentation process. A total of 105 volatile flavour compounds were identified, including 25 alcohols, 8 aldehydes, 7 ketones, 7 acids, 16 esters, 29 terpenes and aromatic compounds, 1 phenol, 1 ether, 1 furan, and 10 other compounds (including alkanes and heterocyclic compounds). Detailed information on all identified volatile flavour compounds and their relative contents at different fermentation stages is provided in Supplementary Table S1. Fig. 1b further illustrates the relative abundance changes of different classes of volatile flavour compounds during fermentation, providing an overview of flavour development in traditional mould-fermented sufu.

**Fig. 1:**
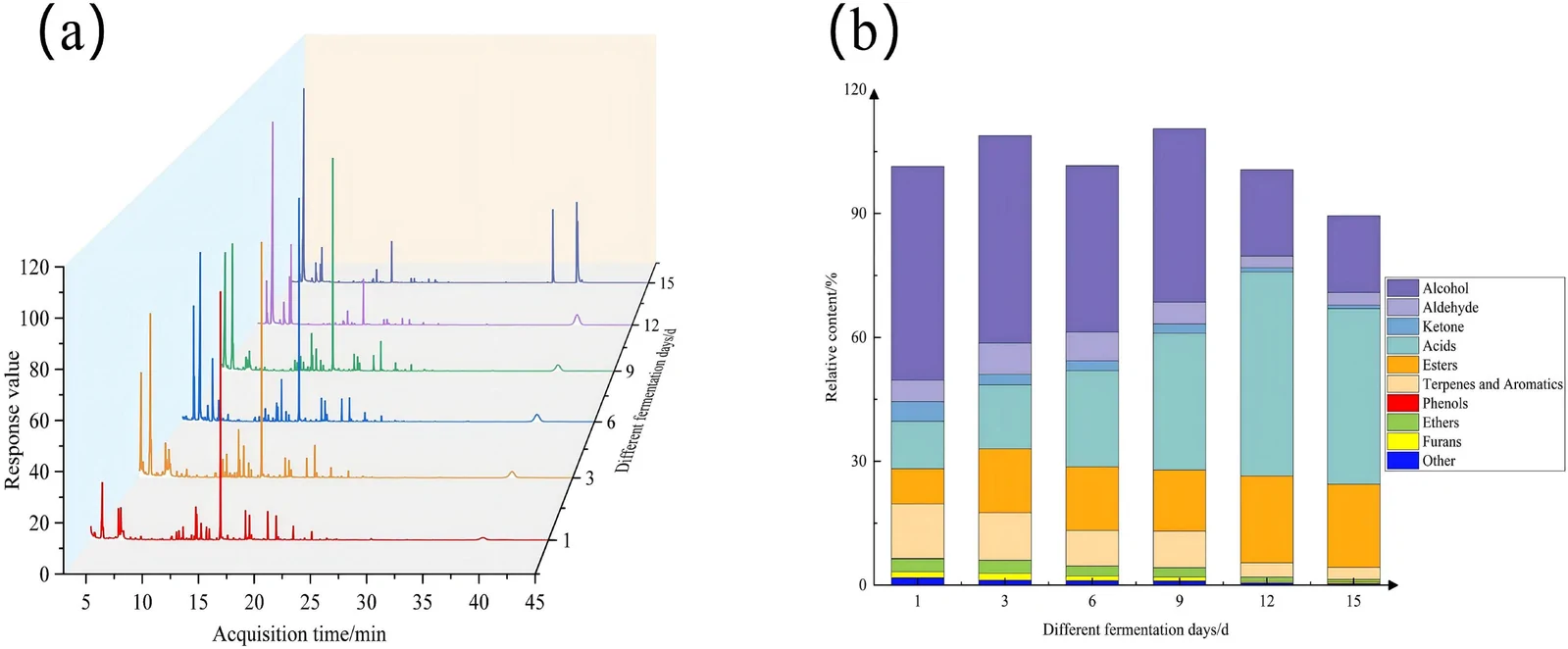
Changes in volatile flavour compounds during traditional mould-fermented sufu fermentation: (a) HS-GC–MS chromatograms at different fermentation stages; (b) stacked plot of the relative contents of volatile flavour compound categories at different fermentation stages.

As shown in Fig. 1b, the composition of volatile flavour compounds in sufu changed markedly throughout fermentation. Alcohol compounds constituted one of the dominant groups of volatile flavour compounds, and their overall content exhibited an initial increase followed by a gradual decline. During the early stage of fermentation, vigorous microbial growth accelerated protein hydrolysis and amino acid degradation, thereby promoting the formation and accumulation of alcohol compounds. In the later stages, some alcohols served as important metabolic intermediates and participated in subsequent reactions, such as esterification, leading to the formation of ester compounds and a corresponding decrease in their relative contents (Bera et al., 2024). Alcohols are important contributors to the characteristic flavour of sufu. For example, phenylethyl alcohol imparts wine-like and rose-like aromas, 1-hexanol contributes a mild fatty note, and linalool provides floral and sweet aromas. Notably, linalool exhibited an initial increase followed by a decrease, whereas 2,3-butanediol increased continuously throughout fermentation. These results suggest that different alcohol compounds may originate from distinct metabolic pathways and perform different roles during flavour formation.

The relative contents of aldehydes and ketones remained comparatively stable throughout fermentation. Aldehydes generally contribute fresh fruity and green aromas (Li et al., 2023) and also serve as important intermediates in lipid oxidation and amino acid metabolism. Ketones are mainly generated through fatty acid degradation and microbial metabolic activities and can impart characteristic dairy-like and fruity notes to sufu. As fermentation progressed, some aldehydes and ketones further participated in condensation, redox, and esterification reactions, contributing to the formation of a more complex flavour profile.

Acid compounds increased continuously throughout fermentation and gradually became one of the dominant groups of volatile flavour compounds. Organic acids, represented primarily by acetic acid, accumulated progressively during fermentation, indicating the continuous conversion of substrates into acidic metabolites through microbial activity. Acid compounds not only contribute characteristic acidic and fermented notes to traditional mould-fermented sufu but also reduce the pH of the fermentation system, thereby inhibiting the growth of undesirable microorganisms and extending product shelf life (Yuan et al., 2024). Furthermore, acids serve as important precursors for ester formation and can promote the accumulation of ester compounds through esterification reactions.

Ester compounds exhibited a progressive increase during fermentation, particularly during the middle and late stages. Esters are important contributors to the characteristic aroma of sufu and are generally associated with fruity and sweet notes (Xu et al., 2025). As fermentation proceeded, alcohols and acids accumulated continuously and underwent esterification reactions under the action of microorganisms and related enzymes, thereby promoting ester formation. The similar increasing trends observed for both acids and esters suggest a close metabolic relationship between these two groups of compounds. Such changes contribute to a more harmonious and well-balanced aroma profile in sufu.

Terpenes and aromatic compounds were present at relatively high levels during the early stages of fermentation but gradually decreased thereafter. These compounds are generally characterized by high volatility and antioxidant activity (Rusu et al., 2023). Enhanced microbial metabolism during the early fermentation stage promoted the release and formation of certain terpenes and aromatic compounds. However, their contents gradually declined during the later stages due to volatilisation losses and further metabolic transformation. For example, 3-carene, β-pinene, and d-limonene all exhibited decreasing trends during fermentation. Owing to their floral, fruity, and citrus-like aroma characteristics, these compounds play important roles in the development of sufu aroma quality.

Phenols, ethers, furans, and other minor compounds were present at relatively low levels throughout fermentation. Although their direct contributions to the overall flavour profile may be limited, some of these compounds possess low odour thresholds and may influence aroma perception through synergistic interactions with other volatile compounds. Furans are often associated with nutty and roasted aroma characteristics, whereas certain heterocyclic and hydrocarbon compounds may participate in the construction of a complex flavour system and provide complementary contributions to the overall flavour profile of sufu.

### 3.2 Identification of potential key aroma-active compounds during Sufu fermentation

The relative contents of volatile flavour compounds obtained by HS-GC–MS do not directly reflect their contributions to the overall aroma profile. Because different compounds possess different odour thresholds, compounds with similar relative contents may contribute differently to aroma perception. Therefore, the relative odour activity value (ROAV) method was employed to screen potential key aroma-active compounds (Luo et al., 2024). ROAV is widely used to evaluate the contribution of individual volatile compounds to the overall aroma of a sample, with the compound showing the greatest contribution assigned a ROAV value of 100. Compounds with ROAV≥1 are generally considered key flavour compounds, whereas compounds with 0.1 ≤ ROAV≤1 are regarded as important aroma modifiers (Xu et al., 2024).

As shown in Table 1, a total of 13 potential key aroma-active compounds (ROAV>1) and six aroma-modifying compounds (0.1 ≤ ROAV≤1) were identified during traditional mould-fermented sufu fermentation. These compounds mainly belonged to alcohols, aldehydes, esters, terpenes and aromatics, and furans, indicating that the characteristic flavour of sufu results from the combined contribution of multiple classes of volatile compounds rather than a single aroma component. During the early fermentation stage, Linalool exhibited the highest ROAV value, suggesting it may be a potential dominant aroma-active compound with prominent floral and sweet notes. As fermentation progressed, 1-Butanol, 3-methyl- gradually became the compound with the highest calculated ROAV value, suggesting that the aroma profile of sufu shifted from a relatively fresh and floral character in the early stage to a more fermented and mellow aroma in the later stages. This flavour succession may be associated with continuous protein and amino acid degradation as well as changes in microbial metabolic activities during fermentation (Chen et al., 2024).

**Table 1:** ROAV values of potential key aroma-active compounds during sufu fermentation.

Serial Number	Compound Name	Odour Threshold(μg/kg)	ROAV						Reference
			1	3	6	9	12	15	
	Alcohol								
1	1-Hexanol	250	0.035	0.058	0.072	0.044	0.052	0.071	(Chen et al., 2019)
2	2-Heptanol	600	0.014	0.014	0.027	0.003	0.003	0.013	(Yang et al., 2021)
3	2-Pentanol	0.29	1.655	3.808	8.716	7.342	20.561	2.276	(Wang et al., 2022)
4	1-Butanol, 3-methyl-	4	74.275	100	100	100	100	100	(Yang et al., 2025)
5	Linalool	6	100	59.378	84.887	85.830	38.592	52.475	(Yang et al., 2025)
6	Phenylethyl Alcohol	45	0.124	0.074	0.098	0.122	0.398	0.147	(Yang et al., 2025)
7	Eucalyptol	1.3	51.654	22.087	29.408	30.886	12.614	16.248	(Hu et al., 2025)
8	1-Octen-3-ol	1	28.019	30.039	29.384	22.814	9.441	20.462	(Hu et al., 2025)
	Aldehyde								
1	Furfural	3000	–	0.001	0.002	0.002	0.001	0.001	(Chen et al., 2019)
2	Heptanal	3	1.530	1.841	2.317	2.129	2.153	2.200	(Li et al., 2024)
3	Benzaldehyde	41.7	0.290	0.196	0.280	0.182	0.226	0.332	(Hu et al., 2025)
4	Benzeneacetaldehyde	4	12.379	14.633	26.856	16.350	20.497	23.927	(Hu et al., 2025)
5	Hexanal	4.5	11.702	17.375	14.042	11.694	4.638	13.935	(Chen et al., 2019)
6	2,4-Heptadienal, (E,E)-	3.5	–	0.379	0.451	0.261	0.710	0.754	(Yang et al., 2025)
	Ketone								
1	3-Octanone	28	0.267	0.276	0.339	0.348	0.178	0.189	(Li et al., 2023)
2	Carvone	50	0.058	0.022	0.044	0.043	–	–	(Li et al., 2023)
	Esters								
1	Butanoic acid, ethyl ester	1	–	–	–	22.205	15.901	31.023	(Wang et al., 2022)
2	Hexanoic acid, ethyl ester	5	–	–	4.297	5.536	3.379	3.828	(Wang et al., 2025)
3	Propanoic acid, 2-hydroxy-, ethylester	150,000	0.001	0.001	0.001	0.001	0.001	0.002	(Wang et al., 2025)
4	Hexadecanoic acid, ethyl ester	2000	0.050	0.073	0.107	0.101	0.384	0.503	(Chen et al., 2019)
	Terpenes and Aromatics								
1	3-Carene	770	0.005	0.038	0.032	0.050	0.017	0.021	(Cheng et al., 2025)
2	alpha.-Pinene	6	–	0.037	0.053	0.051	–	–	(Fu et al., 2025)
3	beta.-Pinene	6	0.926	0.957	0.263	0.646	0.166	0.440	(Fu et al., 2025)
4	d-Limonene	10	3.696	1.635	2.307	2.129	1.391	1.452	(Li et al., 2024)
5	dihydroactinidiolide	0.05	9.662	4.417	12.638	6.084	–	–	(Chen et al., 2023)
6	1,3,6,10-Dodecatetraene, 3,7,11-trimethyl-, (Z,E)-	87	0.006	0.008	0.004	0.014	0.006	–	(Yang et al., 2025)
	Furans								
1	Furan, 2-pentyl-	5.8	6.247	6.360	6.265	5.349	2.827	3.983	(Hu et al., 2025)

Note: “—” indicates that this volatile compound was not present in the sample. Compounds not listed in the table are those for which no relevant odour threshold could be found.

Further analysis indicated that alcohols constituted the largest group of potential key aroma-active compounds, with five alcohols belonging to this group. Among them, 1-Butanol, 3-methyl-, Linalool, and 1-Octen-3-ol maintained relatively high ROAV values throughout multiple fermentation stages, suggesting their potential roles in the formation of the characteristic aroma of sufu. 1-Butanol, 3-methyl- is mainly derived from the catabolism of branched-chain amino acids. Its consistently high ROAV values throughout fermentation suggest that amino acid degradation may represent an important pathway contributing to flavour formation in traditional mould-fermented sufu (Tang et al., 2022). Although fewer aldehydes were identified, Benzeneacetaldehyde and Hexanal maintained relatively high ROAV values across several fermentation stages, suggesting their potential contribution to fruity and fresh aroma characteristics.

In addition, Butanoic acid, ethyl ester and Hexanoic acid, ethyl ester mainly appeared during the middle and late fermentation stages, and their ROAV values gradually increased, suggesting enhanced esterification activity as fermentation progressed. Combined with the simultaneous accumulation of acids and alcohols observed in Section 3.1, it is reasonable to speculate that ester formation may be closely associated with microorganism-mediated esterification reactions. Meanwhile, d-Limonene, dihydroactinidiolide, and Furan, 2-pentyl- also exhibited relatively high ROAV values and were associated with citrus-like, fruity, sweet, and nutty odour descriptors reported in the literature. Together with alcohols, aldehydes, and esters, these compounds collectively constructed the complex and harmonious aroma profile characteristic of traditional mould-fermented sufu. These results further demonstrate that the flavour of sufu is generated through the synergistic effects of multiple potential key aroma-active compounds rather than the contribution of any single aroma component. Moreover, the dynamic changes in these potential key aroma-active compounds provide an important basis for subsequent analyses of the relationships between microbial communities and flavour formation.

### 3.3 Analysis of microbial community structure during Sufu fermentation

#### 3.3.1 Alpha diversity analysis

Alpha diversity reflects species richness and diversity within a single sample (Wu et al., 2023). Common alpha diversity metrics include observed OTU richness, ACE, Chao1, Simpson, and Shannon indices (Zhang et al., 2021). The ACE and Chao1 indices estimate species richness, while the Shannon and Simpson indices measure diversity, with higher values indicating greater community diversity (Li et al., 2026).

The alpha diversity indices for the fungal and bacterial communities during sufu fermentation are presented in Table 2, Table 3, respectively. The alpha diversity indices revealed distinct temporal patterns for fungal and bacterial communities during fermentation. Richness-related indices (OTU, ACE, and Chao1) were generally higher for fungi than for bacteria during the early and middle stages but became higher for bacteria on Day 15. The Simpson index was consistently higher for fungi than for bacteria across all sampling days. For the Shannon index, the bacterial value was slightly higher than the fungal value on Day 1, whereas fungal diversity exceeded bacterial diversity from Day 3 onward. Overall, these results indicate that fungal and bacterial communities followed distinct temporal trajectories in richness and diversity during fermentation, rather than a uniform pattern of fungal dominance throughout the entire process. Additionally, the Coverage values for the sufu samples were close to 1. The higher the Coverage value, the greater the probability that the species present in the sample have been detected, indicating that the sequencing results obtained in this study accurately represent the true microbial composition of the sufu samples.

**Table 2:** Diversity index of fungal communities during sufu fermentation.

Sample	OTU	ACE	Chao1	Simpson	Shannon	Coverage
Day 1 of fermentation	111	111.0000	111.0000	0.8344	3.5758	1.0000
Day 3 of fermentation	119	119.0000	119.0000	0.8247	3.6022	1.0000
Day 6 of fermentation	131	131.3324	131.0000	0.8328	3.5520	1.0000
Day 9 of fermentation	118	119.0670	119.0000	0.8373	3.6916	1.0000
Day 12 of fermentation	96	96.0000	96.0000	0.8438	3.6808	1.0000
Day 15 of fermentation	60	60.0000	60.0000	0.8528	3.7186	1.0000

**Table 3:** Diversity index of bacterial communities during sufu fermentation.

Sample	OTU	ACE	Chao1	Simpson	Shannon	Coverage
Day 1 of fermentation	56	55.0000	55.0000	0.8292	3.6361	1.0000
Day 3 of fermentation	69	70.0958	70.0000	0.6719	2.9940	1.0000
Day 6 of fermentation	73	74.2537	73.7500	0.5644	2.2451	0.9999
Day 9 of fermentation	57	57.3529	57.0000	0.7028	2.5196	1.0000
Day 12 of fermentation	63	63.0000	63.0000	0.7850	3.4197	1.0000
Day 15 of fermentation	154	155.0127	154.3750	0.8032	3.5903	0.9999

A rarefaction curve is constructed by randomly sampling a certain number of sequences from a sample, counting the number of species represented by these sequences, and plotting the relationship between the number of sequences and the number of species. This analysis is used to verify whether the sequencing data volume is sufficient to reflect the species diversity within a sample and indirectly indicates the species richness of the sample (Feng et al., 2024). The rarefaction curves for the fungal and bacterial communities during sufu fermentation are shown in Fig. 2. As can be seen from the figure, for both the fungal and bacterial community rarefaction curves, within a certain range, the curves rose sharply with an increasing number of sequenced reads, indicating a rapid increase in the number of detected species during this phase. As the sequencing depth increased, the curves gradually plateaued, suggesting that the major species present in the samples had been essentially covered, and the sequencing data possessed good representativeness and stability. This further verifies that the data obtained from this experiment can accurately reflect the changes in microbial community diversity during sufu fermentation.

**Fig. 2:**
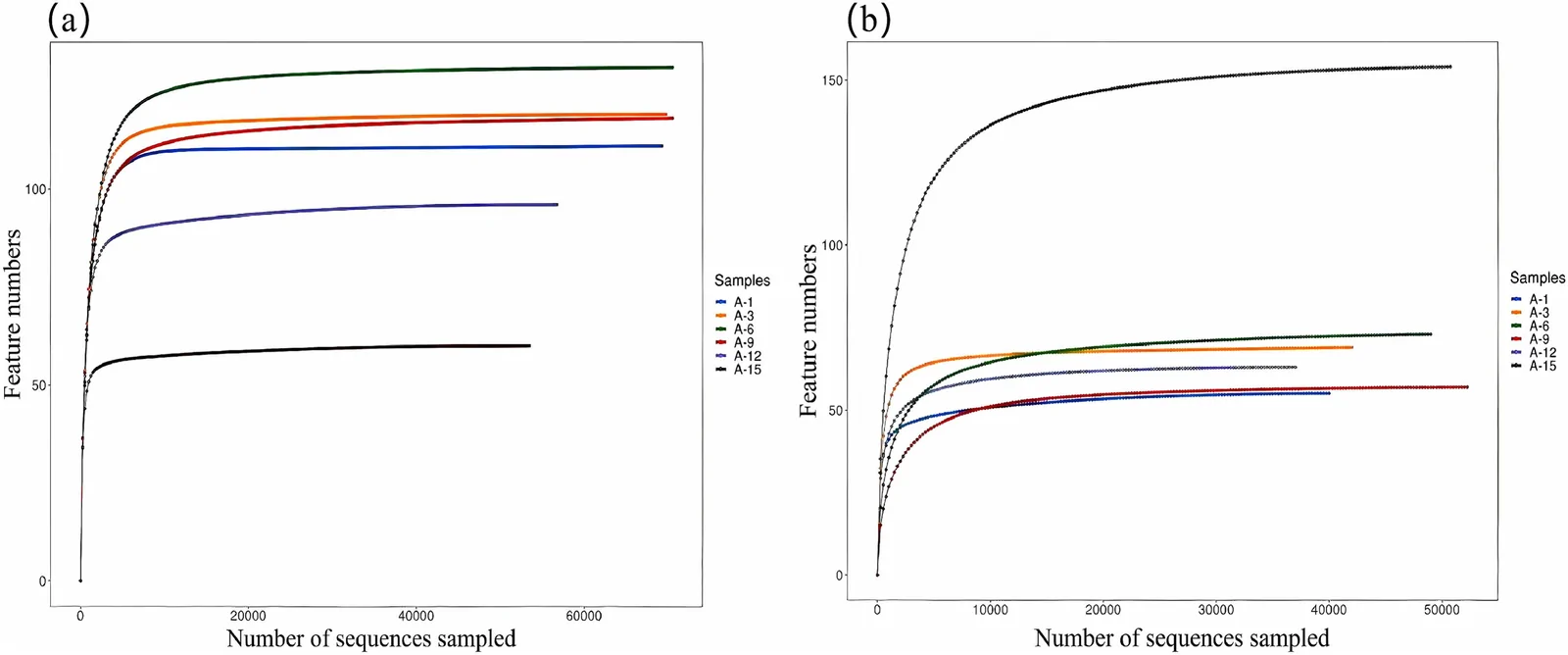
Rarefaction curves for fungal (a) and bacterial (b) communities during sufu fermentation.

#### 3.3.2 Taxonomic annotation statistics

The taxonomic annotation results for the fungal and bacterial communities at different classification levels are presented in Table 4, Table 5, respectively. A total of 6 kingdoms, 42 phyla, 112 classes, 215 orders, 329 families, 410 genera, and 449 species were annotated for the fungal community, while 6 kingdoms, 49 phyla, 70 classes, 169 orders, 248 families, 335 genera, and 442 species were annotated for the bacterial community across all fermentation stages. These results indicate that the sequencing data provided sufficiently detailed taxonomic resolution at multiple classification levels for both microbial groups, supporting the reliability of the community structure analyses presented in the following sections.

**Table 4:** Fungal community structure at different taxonomic levels during sufu fermentation.

Sample	Kingdom	Phylum	Class	Order	Family	Genus	Species
Day 1 of fermentation	1	8	17	34	57	72	81
Day 3 of fermentation	1	7	22	40	60	80	84
Day 6 of fermentation	1	8	21	41	66	80	87
Day 9 of fermentation	1	8	22	38	60	78	86
Day 12 of fermentation	1	5	13	33	54	65	72
Day 15 of fermentation	1	6	17	29	32	35	39

**Table 5:** Bacterial community structure at different taxonomic levels during sufu fermentation.

Sample	Kingdom	Phylum	Class	Order	Family	Genus	Species
Day 1 of fermentation	1	8	11	25	33	39	50
Day 3 of fermentation	1	9	11	27	35	51	67
Day 6 of fermentation	1	9	13	31	40	54	70
Day 9 of fermentation	1	5	7	20	30	41	55
Day 12 of fermentation	1	7	12	27	36	48	61
Day 15 of fermentation	1	11	16	39	74	112	139

#### 3.3.3 Analysis of fungal community structure

The analysis of the fungal community structure at the phylum level during sufu fermentation is presented in Fig. 3. As shown in Fig. 3(a), Ascomycota, Mucoromycota, and Basidiomycota were the predominant phyla throughout the fermentation process, collectively contributing to the biochemical reactions occurring during fermentation. Notably, Ascomycota was the dominant phylum, maintaining an absolute predominance (ranging from 74.62% to 81.96%) over the entire fermentation period. This finding is consistent with the research on the relationship between microbial community succession and flavour formation during the natural fermentation of Hongqu sufu (Luo et al., 2023). The phylum Ascomycota encompasses a large number of fungi with potent enzyme-secreting capabilities, such as Aspergillus and yeasts. These microorganisms secrete abundant hydrolytic enzyme systems, including proteases and lipases, which may catalyse the degradation of soybean proteins into peptides and amino acids (Nakadai et al., 2014). This process has been reported to contribute to the formation of the umami taste and characteristic flavour compounds (such as esters and alcohols) in sufu. Previous studies have demonstrated that the synergistic action of Aspergillus and yeasts can also promote the generation of aromatic substances like ethanol and esters (Zou et al., 2023), significantly enhancing the flavour complexity of the product. The second most abundant phylum was Mucoromycota. Fungi belonging to this phylum possess well-developed mycelia that can penetrate deeply into the tofu matrix, forming a fine mycelial network that imparts a delicate and soft texture to sufu (Xie et al., 2023). Lastly, Basidiomycota, although present in relatively low abundance, may participate in flavour modification and serve complementary functional roles during the fermentation stages. This community structure may contribute to efficient protein and lipid degradation, diverse flavour-compound generation, and the development of characteristic product texture.

**Fig. 3:**
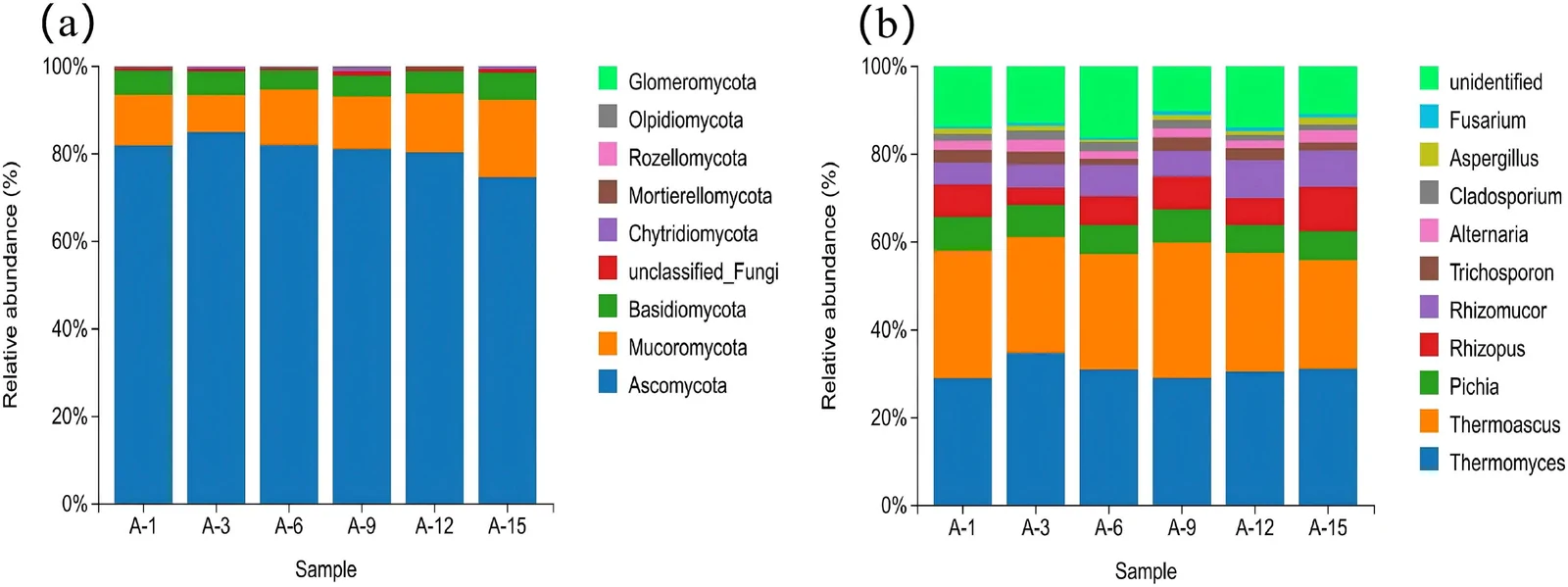
Analysis of fungal community structure during sufu fermentation based on phylum level (a) and genus level (b).

The analysis of the fungal community structure at the genus level during sufu fermentation is presented in Fig. 3. As shown in Fig. 3(b), *Thermomyces* and *Thermoascus* were the dominant genera throughout the fermentation process, maintaining a predominant position (ranging from 26.59% to 28.32%, and from 22.43% to 26.70%, respectively). *Thermomyces* and *Thermoascus* are generally regarded as thermotolerant or thermophilic fungi. However, these genera have also been reported in various fermented food systems under moderate-temperature conditions. Their predominance in the present study may therefore be related to adaptation to the fermentation environment and competitive growth advantages (Nie et al., 2024). These genera are known to produce extracellular hydrolytic enzymes, including proteases and lipases, which contribute to the degradation of proteins and lipids during fermentation. The resulting metabolites may subsequently participate in the formation of volatile flavour compounds such as esters (Yang et al., 2025), potentially contributing to the characteristic mellow flavour and aroma of sufu. This observation was generally consistent with the volatile flavour profile identified by HS-GC–MS, suggesting potential associations between these dominant genera and flavour development during fermentation (He et al., 2019). Previous studies have also reported the involvement of *Thermomyces* and *Thermoascus* in flavour formation during the fermentation of various traditional fermented foods (Zhang et al., 2024). Throughout the entire fermentation process, *Pichia* maintained a relatively stable presence, indicating its adaptability to environmental changes across different fermentation stages. *Pichia* is a potential flavour contributor in sufu; it conducts alcoholic fermentation, producing ethanol (Ruchala et al., 2020), and simultaneously metabolises various aromatic substances, including multiple esters (e.g., ethyl acetate, ethyl caproate). These compounds may collectively contribute to the pleasant winy and fruity notes characteristic of sufu. *Rhizopus* and *Rhizomucor* remained consistently present throughout the process. These genera possess robust mycelia capable of rapid growth on the tofu cubes, forming a dense, white mycelial layer characteristic of mucoraceous fungi. This mycelial network not only imparts the distinctive velvety appearance to sufu but also serves as a natural scaffold, maintaining the intact shape of the tofu cubes while contributing to their soft and delicate texture.

#### 3.3.4 Analysis of bacterial community structure

The analysis of the bacterial community structure at the phylum level during sufu fermentation is presented in Fig. 4. As shown in Fig. 4(a), Proteobacteria and Firmicutes were the predominant phyla throughout the fermentation process, being present across all stages. Among these, Proteobacteria held a dominant position during fermentation (ranging from 89.69% to 81.13%). In the initial stages of fermentation, Proteobacteria can consume oxygen and initiate the decomposition of complex substrates, thereby creating conditions conducive to subsequent fermentation. This finding is consistent with the research conducted by Liang et al. on the microbial communities and physicochemical characteristics of traditional Dajiang and Sufu in North China (Liang et al., 2021). Firmicutes gradually increased during the later stages of fermentation. The lactic acid bacteria within this phylum possess salt-tolerant characteristics, enabling them to remain actively metabolising in high-salt environments (Tian et al., 2023). By producing acids, they may inhibit the growth of spoilage organisms, thereby contributing to product safety, while continuously participating in the decomposition of proteins and lipids, which may promote the accumulation of flavour compounds.

**Fig. 4:**
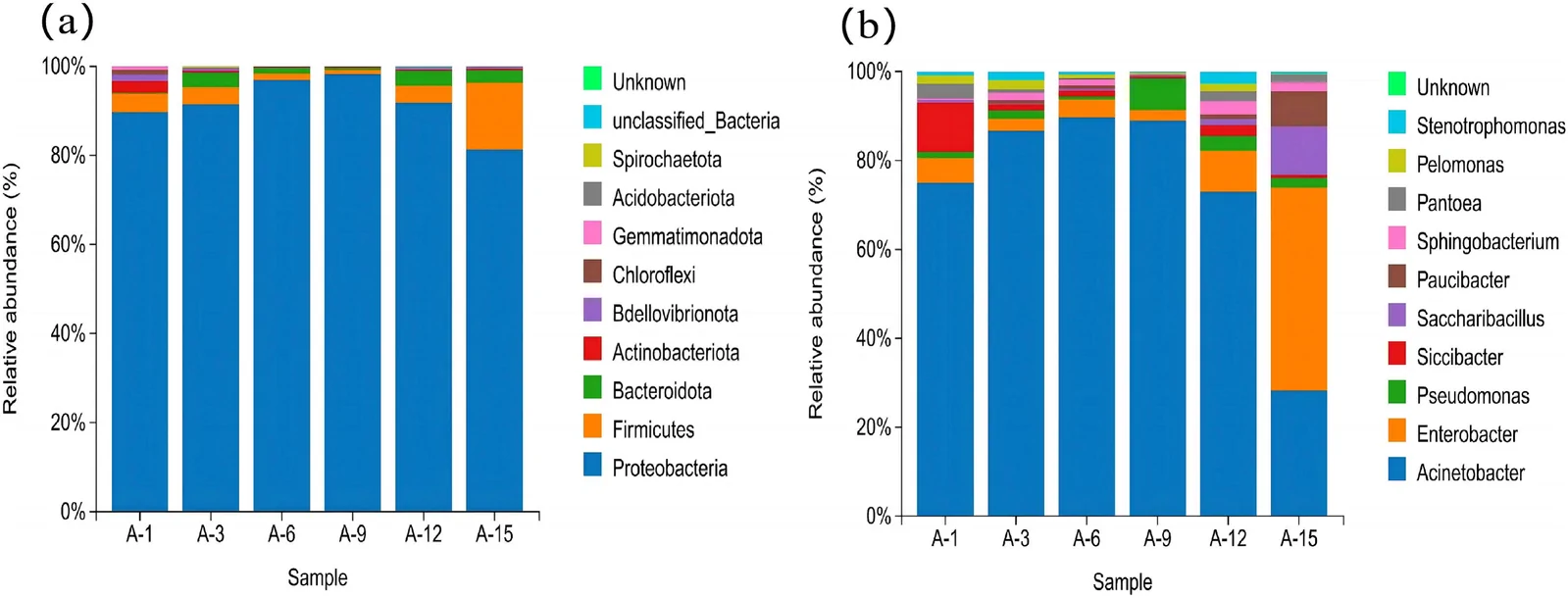
Analysis of bacterial community structure during sufu fermentation based on phylum level (a) and genus level (b).

The analysis of the bacterial community structure at the genus level during sufu fermentation is presented in Fig. 4. As shown in Fig. 4(b), *Acinetobacter*, *Enterobacter*, and *Pseudomonas* were the predominant genera throughout the fermentation process. *Pseudomonas* maintained a relatively stable abundance during the entire fermentation. *Enterobacter* maintained a certain abundance during the early stage of fermentation and gradually became a dominant genus in the later stage, reaching 40.27%. *Acinetobacter* accounted for a relatively high proportion in the early stage of fermentation, being the dominant genus (ranging from 63.08% to 24.93%), and may participate in the initial degradation of proteins during the early fermentation phase. As fermentation progressed, with the accumulation of acidity and the formation of an anaerobic environment, these genera were inhibited, while *Saccharibacillus* gradually emerged. *Saccharibacillus* is reported to produce organic acids (lactic, acetic) that inhibit spoilage organisms and lower pH, potentially promoting protein and fat hydrolysis and subsequent esterification reactions that may generate the fruity, winy esters characteristic of the later fermentation stage. Nevertheless, it should be acknowledged that *Acinetobacter*, *Enterobacter*, and *Pseudomonas* are heterogeneous genera that also encompass members reported to be associated with food spoilage and potential safety risks (e.g., biogenic-amine production or opportunistic pathogenesis) in fermented and other food products (Karwowska et al., 2023; Li et al., 2024). Consequently, although these genera predominated during sufu fermentation, their specific functional roles and safety implications warrant further investigation and experimental validation, and they should not be assumed to contribute solely to flavour formation.

### 3.4 Association analysis between potential key aroma-active compounds and microbial community structure

Volatile flavour compounds identified by HS-GC–MS were screened using relative odour activity values (ROAV) to determine the potential key aroma-active compounds. Pearson correlation analysis was subsequently performed to explore the potential relationships between these potential key aroma-active compounds and microbial communities at both the phylum and genus levels of fungi and bacteria. The results are presented as a correlation heatmap in Fig. 5. In the heatmap, red indicates positive correlations, green indicates negative correlations, and colour intensity reflects the strength of the correlation (Xu et al., 2025). Overall, the heatmap exhibited distinct clustering patterns. Ascomycota, *Pichia*, *Thermoascus*, *Thermomyces*, Proteobacteria, and *Acinetobacter* formed one cluster and showed predominantly positive correlations with most key alcohols, aldehydes, and esters. In contrast, Mucoromycota, Basidiomycota, Firmicutes, Bacteroidota, *Enterobacter*, *Saccharibacillus*, and *Pseudomonas* formed another cluster and were generally negatively correlated with most potential key aroma-active compounds. These results suggest that different microbial taxa may contribute differently to flavour formation during traditional mould-fermented sufu fermentation.

**Fig. 5:**
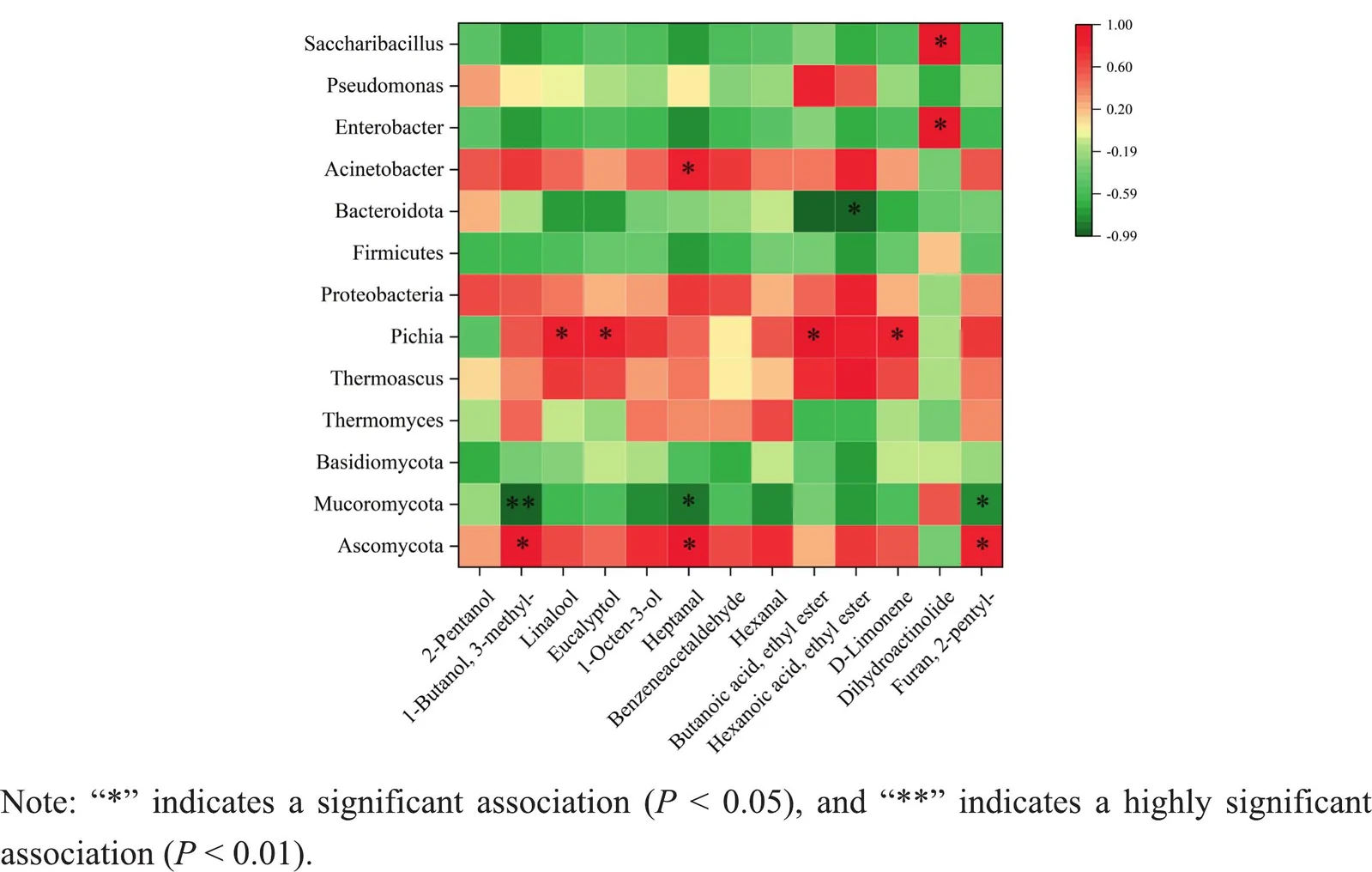
Heatmap of potential key aroma-active compounds and microbial association analysis.

At the fungal phylum and genus levels, Ascomycota, *Pichia*, *Thermoascus*, and *Thermomyces* were generally positively correlated with multiple potential key aroma-active compounds. Among them, Ascomycota showed significant positive correlations with 1-Butanol, 3-methyl-, Heptanal, and Furan, 2-pentyl- (*p* < 0.05), suggesting its potential involvement in the formation of these flavour compounds. *Pichia* was significantly positively correlated with Linalool, Eucalyptol, Butanoic acid, ethyl ester, and d-Limonene (*p* < 0.05), indicating its possible contribution to the accumulation of floral, fruity, and citrus-like aroma compounds. Previous studies have demonstrated that *Pichia* can promote ester formation through esterification reactions, thereby enhancing fruity and floral aromas in fermented foods, which is consistent with the findings reported by Wu (Wu et al., 2024). In contrast, Mucoromycota and Basidiomycota were negatively correlated with most potential key aroma-active compounds. Specifically, Mucoromycota showed a highly significant negative correlation with 1-Butanol, 3-methyl- (*p* < 0.01) and significant negative correlations with Heptanal and Furan, 2-pentyl- (*p* < 0.05), suggesting a potential association with reduced accumulation of these flavour compounds. At the bacterial phylum and genus levels, Proteobacteria and *Acinetobacter* exhibited predominantly positive correlations with alcohols, aldehydes, and esters. *Acinetobacter* showed a significant positive correlation with Heptanal (*p* < 0.05), indicating its possible involvement in the formation and accumulation of aldehyde compounds. *Enterobacter*, *Saccharibacillus*, and *Pseudomonas* were generally negatively correlated with several key alcohols and aldehydes, including 1-Butanol, 3-methyl-, Linalool, Eucalyptol, Benzeneacetaldehyde, and Hexanal. Although *Enterobacter* and *Saccharibacillus* exhibited predominantly negative correlations with several alcohols and aldehydes, both genera showed significant positive correlations with dihydroactinidiolide (*p* < 0.05). Firmicutes and Bacteroidota were negatively correlated with most key volatile flavour compounds. Among them, Bacteroidota showed a significant negative correlation with Hexanoic acid, ethyl ester (*p* < 0.05), suggesting a possible inhibitory effect on ester accumulation.

In summary, the correlation analysis revealed clear association patterns between microbial communities and potential key aroma-active compounds. Positively and negatively correlated microbial groups formed relatively independent association clusters, providing preliminary evidence of microbial involvement in flavour formation during traditional mould-fermented sufu fermentation. These findings provide a theoretical basis for future functional strain screening and validation of flavour formation mechanisms.

## 4 Limitations and future perspectives

This study provides insights into the changes in volatile flavour compounds and microbial communities during traditional sufu fermentation and reveals potential associations between microorganisms and aroma-active compounds. However, several limitations should be acknowledged.

First, volatile flavour compounds were semi-quantified using the GC–MS area normalisation method. Therefore, the calculated ROAV values represent relative estimates of aroma contribution rather than absolute evaluations of aroma impact. Although this approach has been widely used for screening key aroma-active compounds in fermented foods, future studies employing internal standards and absolute quantification would provide more accurate assessments of aroma contributions. In addition, both microbial relative abundance data and volatile compound relative content data are compositional in nature. Therefore, the Pearson association analysis may be affected by closure effects, and the observed associations should be interpreted with caution. Future studies could employ compositional-data analysis approaches, such as centred log-ratio (CLR) transformation or SparCC, or proportionality analysis, to obtain more robust estimates of microbial and flavour associations.

Second, the association analysis between microbial communities and volatile flavour compounds was based on Pearson correlation analysis across six fermentation stages (*n* = 6). The limited sample size reduces the statistical power of the analysis and increases the possibility of false-positive associations. Therefore, the observed relationships should be regarded as exploratory associations rather than evidence of direct causal interactions. Furthermore, multiple-testing correction was not applied during the correlation analysis. As numerous pairwise comparisons were performed, some statistically significant associations may represent false-positive results. Future studies incorporating larger sample sizes, additional biological replicates, and false discovery rate (FDR)-based correction methods are needed to improve the robustness of microbial–flavour association analyses.

In addition, physicochemical factors such as pH, moisture content, salt concentration, and fermentation temperature are known to influence microbial succession and flavour development during fermentation. Although these factors were not included in the present association analysis, they may contribute to the observed variations in microbial communities and volatile flavour compounds. Future studies integrating physicochemical characteristics, microbial dynamics, and flavour profiles will provide a more comprehensive understanding of flavour formation mechanisms during traditional sufu fermentation.

Despite these limitations, the present findings provide a useful basis for identifying potential flavour-related microorganisms and for future validation studies, including inoculation, co-culture fermentation, and flavour reconstitution experiments. Furthermore, these findings offer valuable information for flavour regulation and starter culture development in sufu production.

## 5 Conclusions

HS-GC–MS and high-throughput sequencing were combined to characterise volatile flavour compounds and microbial communities throughout the fermentation cycle of traditional sufu. A total of 105 volatile compounds were detected, of which 13 were identified as potential key aroma-active compounds by ROAV analysis, spanning alcohols (2-pentanol, isoamyl alcohol, linalool, eucalyptol, 1-octen-3-ol), aldehydes (heptanal, benzeneacetaldehyde, hexanal), esters (ethyl butanoate, ethyl hexanoate), terpenes (d-limonene, dihydroactinidiolide), and a furan (2-pentylfuran); the two esters were produced exclusively in the middle and late stages. Fungal communities generally exhibited higher richness and diversity than bacterial communities, although this pattern varied across fermentation stages and diversity metrics. Ascomycota (dominant), Mucoromycota, and Basidiomycota were the leading fungal phyla; *Thermomyces* and *Thermoascus* the leading fungal genera. Proteobacteria and Firmicutes dominated the bacterial community; *Acinetobacter*, *Enterobacter*, and *Pseudomonas* dominated at the genus level. Pearson association analysis revealed potential associations between these communities and potential key aroma-active compounds: Ascomycota, *Thermomyces*, *Thermoascus*, *Pichia*, and Proteobacteria were positively correlated with alcohols, aldehydes, and esters, while Mucoromycota, Basidiomycota, Firmicutes, and Bacteroidota were predominantly negatively correlated. Taken together, these results indicate that the synergistic activity of Ascomycota-affiliated fungi (*Thermomyces*, *Thermoascus*, *Pichia*) and Proteobacteria, rather than either group acting alone, may be associated with the accumulation of key alcohols, aldehydes, and esters during sufu fermentation, while Mucoromycota and Firmicutes appear to play a comparatively limited or even suppressive role in this process. These findings suggest potential synergistic and antagonistic roles of fungi and bacteria in flavour formation and provide a theoretical basis for the targeted regulation of microbial communities and flavour quality improvement in traditional sufu.

• Distinct microbial succession was observed during fermentation, with Ascomycota, *Thermomyces*, and *Thermoascus* dominating the fungal community and Proteobacteria and *Acinetobacter* dominating the bacterial community.
• Pearson analysis revealed potential associations between dominant microbial taxa and potential key aroma-active compounds during fermentation.

## CRediT authorship contribution statement

**Xiangcheng Lu:** Writing – original draft, Methodology, Investigation, Data curation. **Xiaofeng Niu:** Methodology, Investigation, Data curation. **Zhiming Wu:** Methodology, Investigation, Data curation. **Hongmei Zhao:** Validation, Supervision, Resources. **Yanjun Yang:** Supervision, Resources, Methodology. **Xiaoli Ma:** Supervision, Software, Methodology. **Shengli Shi:** Validation, Supervision, Software. **Huihui Wang:** Supervision, Software, Methodology. **Bianfang Hu:** Writing – review & editing, Visualization, Resources, Project administration, Conceptualization. **Aiguo Luo:** Writing – review & editing, Supervision, Resources, Project administration, Funding acquisition, Formal analysis, Conceptualization.

## Funding

This study was supported by “Fund Program for the Scientific Activities of Selected Returned Overseas Professionals in Shanxi Province” (20250038).

## Declaration of competing interest

The authors declare that they have no known competing financial interests or personal relationships that could have appeared to influence the work reported in this paper.

## Data Availability

The 16S rRNA and ITS high-throughput sequencing data generated in this study have been deposited in the NCBI Sequence Read Archive (SRA) under BioProject accession number PRJNA1509546. The remaining data supporting the findings of this study, including the HS-GC–MS volatile compound data, are available from the corresponding author, Aiguo Luo, upon reasonable request.
