# Supplementary material for: Associations between volatile flavour compounds and microbial communities during the fermentation of traditional mould-fermented Sufu
Source: Food Chem X. 2026 Oct 3;39:104552. doi: 10.1016/j.fochx.2026.104552 (PMC13671045; doi:10.1016/j.fochx.2026.104552)
Supplement: Supplementary file 1 [file mmc1.docx]

Supplementary Information

For

Associations Between Volatile Flavour Compounds and Microbial Communities During Traditional Mould-Fermented Sufu Fermentation

This supplementary material provides the complete list of volatile flavour compounds identified by HS-GC-MS during traditional mould-fermented sufu fermentation.

**Table S1.** Volatile flavour compounds identified in traditional mould-fermented sufu at different fermentation stages and their relative contents determined by HS-GC-MS (%)

| serial number | Name of substance | molecular formula | CAS | relative content/% | | | | | |
| --- | --- | --- | --- | --- | --- | --- | --- | --- | --- |
|  |  |  |  | 1 | 3 | 6 | 9 | 12 | 15 |
|  | Alcohol |  |  |  |  |  |  |  |  |
| 1 | 1-Hexanol | C_6_H_14_O | 111-27-3 | 0.36 | 0.66 | 0.57 | 0.36 | 0.26 | 0.27 |
| 2 | 2-Heptanol | C_7_H_16_O | 543-49-7 | 0.35 | 0.39 | 0.52 | 0.05 | 0.03 | 0.12 |
| 3 | 2-Pentanol | C_5_H_12_O | 6032-29-7 | 0.02 | 0.05 | 0.08 | 0.07 | 0.12 | 0.01 |
| 4 | 3-Octanol | C_8_H_18_O | 589-98-0 | - | 0.11 | 0.13 | 0.16 | 0.06 | - |
| 5 | 1-Butanol, 3-methyl- | C_5_H_12_O | 123-51-3 | 12.3 | 18.11 | 12.66 | 13.15 | 8.05 | 6.06 |
| 6 | Linalool | C_10_H_18_O | 78-70-6 | 24.84 | 16.13 | 16.12 | 16.93 | 4.66 | 4.77 |
| 7 | Phenylethyl Alcohol | C_8_H_10_O | 60-12-8 | 0.23 | 0.15 | 0.14 | 0.18 | 0.36 | 0.1 |
| 8 | Cyclobutanol | C_4_H_8_O | 2919-23-5 | - | 0.03 | 0.01 | 0.01 | 0.02 | 0.01 |
| 9 | Eucalyptol | C_10_H_18_O | 470-82-6 | 2.78 | 1.3 | 1.21 | 1.32 | 0.33 | 0.32 |
| 10 | Terpinen-4-ol | C_10_H_18_O | 562-74-3 | - | 1.39 | 1.76 | 1.37 | 0.58 | - |
| 11 | 1-Octen-3-ol | C_8_H_16_O | 3391-86-4 | 1.16 | 1.36 | 0.93 | 0.75 | 0.19 | 0.31 |
| 12 | 2-Nonyn-1-ol | C_9_H_16_O | 5921-73-3 | 0.05 | 0.02 | 0.03 | 0.02 | 0.01 | 0.01 |
| 13 | 2,3-Butanediol | C_4_H_10_O_2_ | 513-85-9 | 0.1 | 0.15 | 0.26 | 2.92 | 3.49 | 5.18 |
| 14 | 1-Butanol, 2-methyl- | C_5_H_12_O | 137-32-6 | - | 2.61 | 2.64 | 2.13 | 1.18 | 0.9 |
| 15 | 2,3-Butanediol, [S-(R*,R*)]- | C_4_H_10_O_2_ | 19132-06-0 | 7.24 | 5.71 | 1.68 | 1.21 | 0.9 | 0.13 |
| 16 | 3-Decyn-2-ol | C_10_H_18_O | 69668-93-5 | - | 0.01 | 0.03 | 0.11 | 0.13 | 0.04 |
| 17 | 2-Heptanol, 3-methyl- | C_8_H_18_O | 31367-46-1 | - | 0.01 | 0.02 | 0.02 | 0.04 | 0.04 |
| 18 | 2-Hexanol, 3-methyl- | C_7_H_16_O | 2313-65-7 | 0.19 | 0.03 | 0.16 | 0.39 | 0.03 | 0.11 |
| 19 | 5-Hexen-2-ol, 5-methyl | C_7_H_14_O | 50551-88-7 | 0.38 | 1.07 | 0.46 | - | - | - |
| 20 | trans-2-Undecen-1-ol | C_11_H_22_O | 75039-84-8 | 0.04 | 0.02 | 0.02 | 0.02 | - | - |
| 21 | Benzeneethanol,.alpha.-methyl | C_9_H_12_O | 698-87-3 | - | - | - | 0.07 | 0.06 | 0.03 |
| 22 | Benzenemethanol,.alpha.,.alpha.,4-trimethyl | C_10_H_14_O | 1197-01-9 | 0.24 | 0.16 | 0.15 | 0.12 | 0.11 | 0.06 |
| 23 | Bicyclo[3.1.0]hexan-2-ol, 2-methyl-5-(1-methylethyl)-, (1.alpha.,2.alpha.,5.alpha.)- | C_10_H_18_O | 17699-16-0 | 1.35 | 0.65 | 0.6 | 0.52 | 0.25 | 0.06 |
| 24 | 3,7-Octadien-2-ol,2-methyl-6- methylene-, (E)- | C_10_H_16_O | 6994-89-4 | 0.08 | 0.09 | 0.04 | 0.09 | 0.07 | 0.01 |
| 25 | (1R,4R)-1-methyl-4-(6-Methylhept-5-en-2-yl)cyclohex-2-enol | C_15_H_26_O | 58334-55-7 | 0.06 | 0.04 | 0.04 | 0.02 | - | - |
|  | Aldehyde |  |  |  |  |  |  |  |  |
| 1 | Furfural | C_5_H_4_O_2_ | 98-01-1 | - | 0.07 | 0.22 | 0.2 | 0.05 | 0.06 |
| 2 | Heptanal | C_7_H_14_O | 111-71-7 | 0.19 | 0.25 | 0.22 | 0.21 | 0.13 | 0.1 |
| 3 | Benzaldehyde | C_7_H_6_O | 100-52-7 | 0.5 | 0.37 | 0.37 | 0.25 | 0.19 | 0.21 |
| 4 | Benzeneacetaldehyde | C_8_H_8_O | 122-78-1 | 2.05 | 2.65 | 3.4 | 2.15 | 1.65 | 1.45 |
| 5 | Hexanal | C_6_H_12_O | 66-25-1 | 2.18 | 3.54 | 2 | 1.73 | 0.42 | 0.95 |
| 6 | Paraldehyde | C_6_H_12_O_3_ | 123-63-7 | 0.18 | 0.44 | 0.47 | 0.56 | 0.14 | 0.09 |
| 7 | Butanal, 3-hydroxy- | C_4_H_8_O_2_ | 107-89-1 | 0.08 | 0.17 | 0.23 | 0.15 | 0.16 | 0.16 |
| 8 | 2,4-Heptadienal,(E,E)- | C_7_H_10_O | 4313-03-5 | - | 0.06 | 0.05 | 0.03 | 0.05 | 0.04 |
|  | Ketone |  |  |  |  |  |  |  |  |
| 1 | 3-Octanone | C_8_H_16_O | 106-68-3 | 0.31 | 0.35 | 0.3 | 0.32 | 0.1 | 0.08 |
| 2 | Carvone | C_10_H_14_O | 99-49-0 | 0.12 | 0.05 | 0.07 | 0.07 | - | - |
| 3 | Xanthoxylin | C_10_H_12_O_4_ | 90-24-4 | 0.48 | 0.14 | 0.14 | 0.11 | 0.19 | 0.14 |
| 4 | 2-Hexanone, 5-methyl | C_7_H_14_O | 110-12-3 | 0.09 | 0.1 | 0.09 | 0.07 | - | - |
| 5 | 5-Hepten-2-one, 4,6-dimethyl | C_9_H_16_O | 31162-48-8 | 0.31 | 0.31 | 0.32 | 0.29 | - | - |
| 6 | 3-Cyclohexen-1-one,3,5,5-trimethyl | C_9_H_14_O | 471-01-2 | 0.34 | 0.1 | 0.04 | 0.04 | 0.02 | - |
| 7 | 3-Cyclohexen-1-one,2-isopropyl-5-methyl | C_10_H_16_O | 1000155-47-0 | 3.12 | 1.49 | 1.43 | 1.38 | 0.74 | 0.62 |
|  | Acids |  |  |  |  |  |  |  |  |
| 1 | Acetic acid | C_2_H_4_O_2_ | 64-19-7 | 11.14 | 15.25 | 22.98 | 32.89 | 49.28 | 42.39 |
| 2 | Alanine | C_3_H_7_NO_2_ | 56-41-7 | 0.01 | 0.04 | 0.01 | 0.01 | 0.02 | 0.01 |
| 3 | D-Alanine | C_3_H_7_NO_2_ | 338-69-2 | 0.13 | 0.02 | 0.04 | 0.06 | 0.03 | 0.03 |
| 4 | 2-Nonynoic acid | C_9_H_14_O_2_ | 1846-70-4 | 0.05 | 0.03 | 0.05 | 0.01 | 0.01 | 0.01 |
| 5 | 2-Octynoic acid | C_8_H_12_O_2_ | 5663-96-7 | 0.11 | 0.14 | 0.11 | 0.07 | 0.03 | 0.05 |
| 6 | Heptanoic acid, 2-ethyl | C_9_H_18_O_2_ | 3274-29-1 | - | - | 0.07 | 0.04 | 0.02 | 0.02 |
| 7 | 1,2,4-Benzenetricarboxylic acid | C_11_H_10_O_6_ | 54699-35-3 | 0.06 | 0.03 | 0.03 | 0.02 | - | - |
|  | Esters |  |  |  |  |  |  |  |  |
| 1 | Butanoic acid, ethyl ester | C_6_H_12_O_2_ | 105-54-4 | - | - | - | 0.73 | 0.32 | 0.47 |
| 2 | Hexanoic acid, ethyl ester | C_8_H_16_O_2_ | 123-66-0 | - | - | 0.68 | 0.91 | 0.34 | 0.29 |
| 3 | Propanoic acid, 2-hydroxy-,ethylester | C_5_H_10_O_3_ | 97-64-3 | 3.85 | 7.75 | 6.7 | 5.08 | 4.24 | 3.45 |
| 4 | Butanoic acid, 3-methyl-, 1-ethenyl-1,5-dimethyl-4-hexenyl ester | C_15_H_26_O_2_ | 1118-27-0 | - | 0.12 | 0.08 | 0.11 | - | - |
| 5 | Benzoic acid, ethyl ester | C_9_H_10_O_2_ | 93-89-0 | - | 0.05 | 0.07 | 0.12 | 0.04 | 0.06 |
| 6 | Tetradecanoic acid,ethyl ester | C_16_H_32_O_2_ | 124-06-1 | - | 0.15 | 0.23 | 0.27 | 0.16 | 0.17 |
| 7 | Dodecanoic acid,ethyl ester | C_14_H_28_O_2_ | 106-33-2 | - | 0.09 | 0.15 | 0.12 | 0.04 | 0.05 |
| 8 | Hexadecanoic acid,ethyl ester | C_18_H_36_O_2_ | 628-97-7 | 4.15 | 6.62 | 6.77 | 6.64 | 15.45 | 15.23 |
| 9 | Myrtenyl formate | C_11_H_16_O_2_ | 1000430-69-7 | 0.02 | 0.02 | 0.01 | 0.01 | - | - |
| 10 | Lilac alcohol formate A | C_11_H_18_O_3_ | 1010141-22-9 | 0.13 | 0.11 | 0.14 | 0.14 | 0.06 | 0.03 |
| 11 | Butanoic acid, 2-methyl-, ethyl ester | C_7_H_14_O_2_ | 7452-79-1 | - | 0.07 | 0.08 | 0.09 | - | - |
| 12 | 4-Terpinenyl acetate | C_12_H_20_O_2_ | 4821-04-9 | 0.34 | 0.26 | 0.22 | 0.23 | 0.28 | 0.29 |
| 13 | trans-Pinocarvyl acetate | C_12_H_18_O_2_ | 1686-15-3 | 0.02 | 0.01 | 0.02 | 0.03 | - | - |
| 14 | 1-Butanol, 3-methyl-, acetate | C_7_H_14_O_2_ | 123-92-2 | - | 0.12 | 0.12 | 0.14 | - | - |
| 15 | Pentanoic acid,2,2,4-trimethyl-3-hydroxy-, isobutyl ester | C_12_H_24_O_3_ | 244074-78-0 | 0.02 | 0.01 | 0.01 | - | - | - |
| 16 | 8-Methylnonanoic acid, ethyl ester | C_12_H_24_O_2_ | 1000452- 00-8 | - | 0.08 | 0.16 | 0.16 | 0.07 | 0.1 |
|  | Terpenes and Aromatics |  |  |  |  |  |  |  |  |
| 1 | Amorpha-4,7(11)-diene | C_15_H_24_ | 486998-53-2 | 0.19 | 0.1 | 0.11 | 0.12 | 0.04 | - |
| 2 | 3-Carene | C_10_H_16_ | 13466-78-9 | 0.17 | 1.31 | 1.07 | 1.27 | 0.26 | 0.24 |
| 3 | (+)-4-Carene | C_10_H_16_ | 29050-33-7 | 0.31 | 0.35 | 0.34 | 0.17 | 0.11 | 0.14 |
| 4 | alpha.-Pinene | C_10_H_16_ | 80-56-8 | - | 0.01 | 0.01 | 0.01 | - | - |
| 5 | beta.-Pinene | C_10_H_16_ | 127-91-3 | 0.23 | 0.26 | 0.13 | 0.21 | 0.02 | 0.04 |
| 6 | Naphthalene,1,2,3,5,6,8a hexahydro-4,7-dimethyl-1-(1-methylethyl)-, (1S cis)- | C_15_H_24_ | 483-76-1 | 0.14 | 0.07 | 0.07 | 0.08 | 0.03 | 0.03 |
| 7 | D-Limonene | C_10_H_16_ | 5989-27-5 | 1.53 | 0.74 | 0.73 | 0.7 | 0.28 | 0.22 |
| 8 | Germacrene D | C_15_H_24_ | 23986-74-5 | 0.05 | 0.02 | 0.02 | 0.03 | 0.01 | 0.07 |
| 9 | Naphthalene,decahydro-4a-methyl-1-methylene-7-(1-methylethenyl)-,[4aR-(4a.alpha.,7.alpha.,8a.beta.)]- | C_15_H_24_ | 17066-67-0 | 0.25 | 0.19 | 0.15 | 0.16 | 0.05 | - |
| 10 | o-Menth-8-ene | C_10_H_18_ | 15193-25-6 | 0.04 | 0.02 | 0.02 | 0.02 | - |  |
| 11 | Sabinol,trans- | C_10_H_16_O | 29606-76-6 | - | 0.08 | 0.17 | 0.1 | - | - |
| 12 | 1,3,5-Cycloheptatriene | C_7_H_8_ | 544-25-2 | 0.1 | 0.15 | 0.12 | 0.15 | 0.06 | 0.04 |
| 13 | Epizonarene | C_15_H_24_ | 41702-63-0 | 0.08 | 0.03 | 0.03 | 0.04 | 0.01 |  |
| 14 | p-Cymene | C_10_H_14_ | 99-87-6 | 0.44 | 0.38 | 0.22 | 0.26 | 0.06 | 0.07 |
| 15 | m-Aminophenylacetylene | C_8_H_7_N | 54060-30-9 | 0.48 | 0.48 | 0.27 | 0.15 | 0.16 | 0.2 |
| 16 | dihydroactinidiolide | C_11_H_16_O_2_ | 15356-74-8 | 0.02 | 0.01 | 0.02 | 0.01 | - | - |
| 17 | 1,3,6,10-Dodecatetraene,3,7,11-trimethyl-,(Z,E)- | C_15_H_24_ | 26560-14-5 | 0.02 | 0.03 | 0.01 | 0.04 | 0.01 | - |
| 18 | Megastigma-4,6(Z),8(Z)-triene | C_13_H_20_ | 71186-25-9 | 0.11 | 0.08 | 0.07 | 0.08 | 0.03 | - |
| 19 | 3-Methyl-trans-3a,4,7,7atetrahydroindane | C_10_H_16_ | 1000145-84-3 | 2.52 | 1.27 | 1.49 | 1.17 | 0.59 | 0.43 |
| 20 | Cyclohexene, 3-methyl-6-(1- methylethylidene)- | C_10_H_16_ | 586-63-0 | 0.15 | 0.07 | 0.06 | 0.05 | 0.01 | 0.04 |
| 21 | Bicyclo[3.1.1]hept-2-ene, 3,6,6- trimethyl | C_10_H_16_ | 4889-83-2 | - | 0.15 | 0.08 | 0.11 | - | - |
| 22 | 1,3-Cyclohexadiene,1-methyl-4-(1-methylethyl)- | C_10_H_16_ | 99-86-5 | 0.52 | 0.35 | 0.3 | 0.33 | 0.84 | 0.45 |
| 23 | Cyclohexene, 1-methyl-5-(1- methylethenyl)-,(R)- | C_10_H_16_ | 1461-27-4 | 3.41 | 3.41 | 1.71 | 1.99 | 0.45 | 0.61 |
| 24 | Bicyclo[2.2.1]heptane, 7,7-dimethyl-2-methylene | C_10_H_16_ | 471-84-1 | 0.02 | 0.02 | 0.01 | 0.01 | - | - |
| 25 | Bicyclo[3.1.0]hexane, 4-methylene-1-(1-methylethyl)- | C_10_H_16_ | 3387-41-5 | 0.85 | 1.08 | 0.5 | 0.63 | 0.13 | 0.16 |
| 26 | Tricyclo[5.4.0.0(2,8)]undec-9-ene,2,6,6,9-tetramethyl-, (1R,2S,7R,8R)- | C_15_H_24_ | 5989-08-2 | 0.94 | 0.51 | 0.49 | 0.54 | 0.19 | 0.13 |
| 27 | Bicyclo[7.2.0]undec-4-ene, 4,11,11-trimethyl-8-methylene-,[1R-(1R*,4Z,9S*)]- | C_15_H_24_ | 118-65-0 | 0.13 | 0.07 | 0.07 | 0.08 | - | - |
| 28 | (1S,4aR,8aS)-1-Isopropyl-7-methyl-4-methylene-1,2,3,4,4a,5,6,8a octahydronaphthalene | C_15_H_24_ | 6980-46-7 | 0.28 | 0.15 | 0.17 | 0.18 | 0.07 | 0.04 |
| 29 | Naphthalene,1,2,4a,5,6,8a hexahydro-4,7-dimethyl-1-(1-methylethyl)- | C_15_H_24_ | 483-75-0 | 0.19 | 0.11 | 0.11 | 0.14 | 0.05 | 0.01 |
|  | Phenols |  |  |  |  |  |  |  |  |
| 1 | p-Cumenol | C_9_H_12_O | 99-89-8 | 0.15 | 0.05 | 0.07 | 0.06 | 0.03 | - |
|  | Ethers |  |  |  |  |  |  |  |  |
| 1 | Estragole | C_10_H_12_O | 140-67-0 | 3.06 | 3.12 | 2.38 | 2.2 | 1.06 | 0.66 |
|  | Furans |  |  |  |  |  |  |  |  |
| 1 | Furan, 2-pentyl- | C_9_H_14_O | 3777-69-3 | 1.5 | 1.67 | 1.15 | 1.02 | 0.33 | 0.35 |
|  | Other (including alkanes, heterocycles, etc.) |  |  |  |  |  |  |  |  |
| 1 | 1,3-Cycloheptadiene | C_7_H_10_ | 4054-38-0 | 0.09 | 0.12 | 0.08 | 0.1 | 0.04 | - |
| 2 | 2,3-Epoxybutane | C_4_H_8_O | 3266-23-7 | 0.16 | 0.1 | 0.09 | 0.09 | 0.09 | 0.11 |
| 3 | 1-Butene, 4-methoxy | C_5_H_10_O | 4696-30-4 | 0.01 | 0.01 | 0.04 | 0.01 | 0.04 | 0.03 |
| 4 | 1,3,6-Heptatriene,5-methyl | C_8_H_12_ | 925-52-0 | 0.16 | 0.12 | 0.06 | 0.09 | 0.04 | - |
| 5 | (S)-(+)-1-Cyclohexylethylamine | C_8_H_17_N | 17430-98-7 | 0.04 | 0.05 | 0.02 | 0.02 | 0.03 | 0.05 |
| 6 | 1,5,9-Cyclododecatriene,1-methyl-, (E,E,E)- | C_13_H_20_ | 15782-27-1 | 0.26 | 0.15 | 0.17 | 0.18 | 0.06 | 0.03 |
| 7 | 2-Methyl-3-vinyl oxirane | C_5_H_8_O | 6790-37-0 | 0.27 | 0.36 | 0.26 | 0.2 | 0.11 | 0.07 |
| 8 | 9-Oxabicyclo[6.1.0]non-3-ene | C_8_H_12_O | 38202-42-5 | 0.01 | 0.03 | 0.01 | 0.03 | - | - |
| 9 | Tricyclo[2.2.1.0(2,6)]heptane, 1,7,7- trimethyl | C_10_H_16_ | 508-32-7 | 0.61 | 0.22 | 0.32 | 0.26 | 0.15 | 0.11 |
| 10 | 1,3-Cyclopentadiene, 5-(1,3-dimethylbutylidene)- | C_11_H_16_ | 7338-49-0 | 0.18 | 0.06 | 0.03 | 0.02 | 0.01 | - |

Note: “–” indicates that the compound was not detected in the corresponding sample.
